# Genomic analysis and antimicrobial resistance of *Neisseria gonorrhoeae* isolates from Vietnam in 2011 and 2015–16

**DOI:** 10.1093/jac/dkaa040

**Published:** 2020-02-18

**Authors:** Pham Thi Lan, Daniel Golparian, Johan Ringlander, Le Van Hung, Nguyen Van Thuong, Magnus Unemo

**Affiliations:** d1 Hanoi Medical University, National Hospital of Dermatology and Venereology, Hanoi, Vietnam; d2 WHO Collaborating Centre for Gonorrhoea and Other Sexually Transmitted Infections, Swedish Reference Laboratory for STIs, Faculty of Medicine and Health, Örebro University, Örebro, Sweden; d3 Department of Infectious Diseases at Institute of Biomedicine, University of Gothenburg, Gothenburg, Sweden

## Abstract

**Objectives:**

Antimicrobial resistance (AMR) in *Neisseria gonorrhoeae*, compromising gonorrhoea treatment, is a threat to reproductive health globally. South-East and East Asia have been major sources of emergence and subsequent international spread of AMR gonococcal strains during recent decades. We investigated gonococcal isolates from 2011 and 2015–16 in Vietnam using AMR testing, WGS and detection of AMR determinants.

**Methods:**

Two hundred and twenty-nine gonococcal isolates cultured in 2015–16 (*n = *121) and 2011 (*n = *108) in Vietnam were examined. AMR testing was performed using Etest and WGS with Illumina MiSeq.

**Results:**

Resistance among the 2015–16 isolates was as follows: ciprofloxacin, 100%; tetracycline, 79%; benzylpenicillin, 50%; cefixime, 15%; ceftriaxone, 1%; spectinomycin, 0%; and 5% were non-WT to azithromycin. Eighteen (15%) isolates were MDR. The MIC range for gentamicin was 2–8 mg/L. Among the 2015–16 isolates, 27% (*n = *33) contained a mosaic *penA* allele, while no isolates had a mosaic *penA* allele in 2011. Phylogenomic analysis revealed introduction after 2011 of two mosaic *penA*-containing clones (*penA*-10.001 and *penA*-34.001), which were related to cefixime-resistant strains spreading in Japan and Europe, and a minor clade (eight isolates) relatively similar to the XDR strain WHO Q.

**Conclusions:**

From 2011 to 2015–16, resistance in gonococci from Vietnam increased to all currently and previously used antimicrobials except ceftriaxone, spectinomycin and tetracycline. Two mosaic *penA-*containing clones were introduced after 2011, explaining the increased cefixime resistance. Significantly increased AMR surveillance, antimicrobial stewardship and use of WGS for molecular epidemiology and AMR prediction for gonococcal isolates in Vietnam and other Asian countries are crucial.

## Introduction

*Neisseria gonorrhoeae* has developed antimicrobial resistance (AMR) to all drugs used in the treatment of gonorrhoea using mainly all known AMR mechanisms.^1–3^ The extended-spectrum cephalosporin (ESC) ceftriaxone is the last option for first-line empirical monotherapy; however, gonococcal resistance to ceftriaxone and occasional treatment failures with ceftriaxone have been verified.^3^ Ceftriaxone (250–500 mg) plus azithromycin (1–2 g orally) is the empirical first-line treatment in most high-income countries, but some countries recommend high-dose (1 g) ceftriaxone monotherapy, i.e. when chlamydia has been excluded.^4–9^ Worryingly, international spread of a ceftriaxone-resistant strain (FC428), initially identified in Japan,^10^ has been verified since 2015.^11–18^ The first gonorrhoea treatment failure with ceftriaxone plus azithromycin was reported in 2016,^19^ and in 2018 the first gonococcal strain with ceftriaxone resistance combined with high-level azithromycin resistance was identified in the UK and Australia (WHO Q).^20–22^ This development emphasizes the urgent need for new antimicrobials for gonorrhoea treatment^23^ and increased AMR surveillance globally.^2^^,^^3^

During the past several decades, South-East and East Asia have had high gonorrhoea prevalences and been major sources of emergence and subsequent international spread of gonococcal AMR.^3^^,^^24–26^ Most of the ceftriaxone-resistant cases during recent years have been associated with travel to South-East or East Asia.^3^^,^^10–22^ In Vietnam, quality-assured surveillance of gonococcal AMR has been very limited, and non-prescribed antimicrobials are widely available over the counter.^27^ The recommended treatment for uncomplicated gonorrhoea in Vietnam is 400 mg of cefixime, 250 mg of ceftriaxone, 2 g of spectinomycin or 1 g of cefotaxime, and all these are given together with 200 mg of doxycycline daily for 7 days.^28^ Worryingly, one recent study reported exceedingly high rates of gonococcal AMR, including 5% ceftriaxone resistance, in 2011 in Hanoi, Vietnam.^27^

WGS, due to its high resolution, is ideal for enhanced understanding of the gonococcal population, for molecular epidemiology of AMR gonococcal strains and to detect AMR determinants and predict AMR in gonococci nationally and internationally.^29–35^ However, WGS has been conducted on few gonococcal isolates from Asia, and no WGS of isolates from Vietnam has been published.

Our aims were to investigate: (i) resistance to previously and currently used gonorrhoea therapeutic antimicrobials in gonococcal isolates cultured in Vietnam in 2015–16 compared with 2011;^27^ and (ii) phylogenomics and AMR determinants among all these isolates using WGS.

## Materials and methods

### N. gonorrhoeae isolates

Two-hundred and twenty-nine clinical gonococcal isolates from Hanoi, Vietnam were examined. Of these, 121 isolates were cultured in 2015–16 from urethral swabs from 117 males and cervical swabs from four females. The mean (median) age was 30 (29) years for males (range: 16–61 years) and 28 (26) years for females (range: 23–34 years). All patients were diagnosed and treated at the National Hospital of Dermatology and Venereology, Hanoi, Vietnam. Additionally, 108 isolates cultured in the same setting in 2011 and previously published with regard to AMR^27^ were included in the WGS analysis. All isolates from 2011 and 2015–16 were cultured and preserved as part of the routine diagnostics in Vietnam. No patient identification data, except age and gender, were available; accordingly, ethical approval was not required. Prior to AMR testing and WGS, all isolates were cultured on Difco GC Medium Base agar (BD, Diagnostics, Sparks, MD, USA) supplemented with 1% haemoglobin (BD), 10% horse serum and 1% IsoVitalex (BD).

The 2016 WHO F gonococcal reference strain^36^ was used as reference for mapping and was excluded prior to building the phylogenomic trees. For comparison, the internationally spreading ceftriaxone-resistant strain FC428^10^ and WHO Q with ceftriaxone resistance and high-level azithromycin resistance, identified in 2018 and associated with travel to South-East Asia,^20–22^ were included in the WGS analysis. All Vietnamese isolates were compared with previously published cefixime-resistant isolates from Japan^37^ (*n = *69; 2015) and 11 European countries^32^ (*n = *42; 2013). The 2016 WHO gonococcal reference strains^36^ were used for phenotypic and genetic quality controls.

### AMR testing

AMR testing for ceftriaxone, cefixime, azithromycin, spectinomycin, ciprofloxacin, benzylpenicillin, tetracycline and gentamicin was performed using the Etest (bioMérieux, Marcy-l’Étoile, France). MICs (mg/L) were interpreted according to the EUCAST clinical breakpoints,^38^ where available. For azithromycin, the EUCAST azithromycin epidemiological cut-off (ECOFF) of 1 mg/L (www.eucast.org/clinical_breakpoints) was used to identify non-WT isolates for azithromycin. Additionally, an ESC MIC of 0.125 mg/L was considered as decreased ESC susceptibility because isolates with this MIC have been associated with ESC treatment failures.^3^^,^^25^

### WGS

DNA was extracted from bacterial suspensions using a customized QIAsymphony (QIAGEN GmbH, Hilden, Germany) DNA extraction protocol. Quality controls of the extracted DNA and the Nextera XT libraries (Illumina, Inc., San Diego, CA, USA) were performed using Qubit (Thermo Fisher Scientific, Waltham, MA, USA) and Tapestation (Agilent Technologies, Santa Clara, CA, USA).

Sequencing libraries were prepared using the Nextera XT DNA library preparation kit (Illumina, Inc.) and WGS was performed using Illumina MiSeq (Illumina, Inc.), as previously described.^31^ Illumina reads were quality controlled, Illumina adaptors removed, and reads were trimmed according to Phred quality score Q30, mapped to different references for additional contamination control and assembled; and AMR determinants and genes included in the MLST and *N. gonorrhoeae* multiantigen sequence typing (NG-MAST) schemes were extracted using a customized CLC Genomics Workbench (v9.5.3) (QIAGEN GmbH) workflow (Figure S1, available as Supplementary data at *JAC* Online)*.* The frequency of 23S rRNA allele mutations was identified using mapping and quality-based variant detection (Neighborhood Quality Standard algorithm).^36^ Reads from all isolates were mapped to the genome sequence of the WHO F gonococcal reference strain^36^ using Burrows–Wheeler Aligner (BWA) v0.7.17,^39^ SNPs were called and recombinant regions were removed using Gubbins v1.4.10.^40^ Subsequently, a maximum-likelihood phylogenetic tree based on inherited SNPs was obtained using RAxML v8.2.8.^41^ All Vietnamese isolates were compared with cefixime-resistant isolates from Japan^37^ and Europe^32^ in a phylogenetic tree created as described above without removing the recombinant regions and visualized in Microreact.^42^

All WGS sequence reads of the Vietnamese isolates are available from the ENA (PRJEB34425).

## Results

### AMR

*In vitro* resistance among the 2015–16 isolates (*n = *121) was as follows: ciprofloxacin, 100% (*n = *121); tetracycline, 79% (*n = *95); benzylpenicillin, 50% (*n = *60); cefixime, 15% (*n = *18); ceftriaxone, 1% (*n = *1); spectinomycin, 0% (*n = *0); and 5% (*n = *6) were non-WT to azithromycin (Table 1). Fifteen percent (*n = *18) of the isolates were MDR.^43^ For gentamicin, the MIC range (2–8 mg/L), MIC_50_ (4 mg/L) and MIC_90_ (8 mg/L) showed high *in vitro* susceptibility. The AMR results for the isolates from 2011 have been previously published.^27^ Briefly, from 2011 to 2015–16, resistance to all examined antimicrobials except ceftriaxone, spectinomycin and tetracycline increased (Table 1). One (0.8%) isolate in 2015–16 was resistant to ceftriaxone compared with five (4.6%) isolates in 2011.^27^ Furthermore, 15 (12.4%) of the 2015–16 isolates had a decreased susceptibility to ceftriaxone compared with 25 (23.1%) isolates in 2011. In contrast, 18 (14.9%) isolates from 2015–16 were resistant to cefixime compared with only 1 isolate (0.9%) from 2011,^27^ which was the only XDR^43^ isolate in this study. Additionally, 12.4% of the 2015–16 isolates had a decreased susceptibility to cefixime compared with only 7.4% of the isolates from 2011,^27^ and the MIC_90_ of cefixime had increased from 0.064 mg/L in 2011 to 0.25 mg/L in 2015–16.

**Table 1.** Antimicrobial susceptibility of *N. gonorrhoeae* isolates cultured in Vietnam in 2011 (*n *=* *108)^27^ and 2015–16 (*n *=* *121)

Antimicrobial (breakpoints, mg/L)	MIC range 2011/2015–16	MIC_50_ 2011/2015–16	MIC_90_ 2011/2015–16	S/I (D)/R % 2011	S/I (D)/R % 2015–16
Cefixime (S <0.125, R >0.125)	<0.016 to 0.25/<0.016 to 0.5	0.032/0.064	0.125/0.25	92/7/1	73/12/15
Ceftriaxone (S <0.125, R >0.125)	<0.002 to 0.25/0.004 to 0.25	0.064/0.064	0.125/0.064	72/23/5	87/12/1
Azithromycin (non-WT >1)	0.032 to 4/0.032 to 2	0.25/0.5	1/1	97/NA/3	95/NA/5
Spectinomycin (S ≤64, R >64)	4 to 16/8 to 32	16/16	16/16	100/NA/0	100/NA/0
Ciprofloxacin (S ≤0.032, R >0.064)	0.008 to >32/0.5 to >32	>32/24	>32/>32	2/0/98	0/0/100
Tetracycline (S ≤0.5, R >1)	0.25 to >256/0.75 to >256	32/4	128/256	6/12/82	0/21/79
Benzylpenicillin (S ≤0.064, R >1)	0.064 to >32/0.25 to >32	1/1	>32/>32	2/51/47	0/50/50
Gentamicin (NA)	0.032 to 8/2 to 8	4/4	4/8	NA	NA

S, susceptible; I (D), intermediate (decreased); R, resistant; NA, not applicable.

### Molecular epidemiology, AMR determinants and phylogenomics

**Figure 1.**
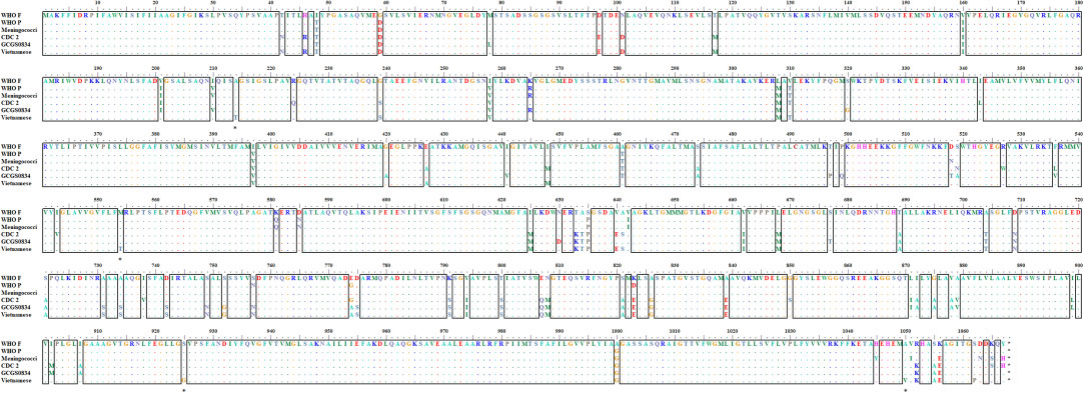
Amino acid sequences of MtrD in WHO F (azithromycin susceptible)^36^ and WHO P (azithromycin non-WT)^36^ and a meningococcal MtrD sequence (included as reference). GCGS0834^45^ and CDC 2^46^ were included as the two previously described mosaic MtrD sequences most similar to the Vietnamese MtrD sequence, which had only four unique amino acid alterations (asterisks). Conserved regions are boxed. This figure appears in colour in the online version of *JAC* and in black and white in the print version of *JAC*.

The genomic heterogeneity among all isolates (*n *=* *229) was large (Figure 1). One hundred and nineteen NG-MAST STs were identified; 52 new STs and 64 represented by single isolates. The most common NG-MAST STs were ST4787 (*n *=* *14), ST7741 (*n *=* *12), ST9666 (*n *=* *9), ST7720 (*n *=* *7) and ST5061 (*n *=* *5). Forty MLST STs, including two new STs and 12 STs represented by single isolates, were found. The most common MLST STs were ST7371 (*n *=* *28), ST1588 (*n *=* *22), ST1600 (17), ST7363 (*n *=* *17) and ST1901 (*n *=* *15).

A mosaic *penA* allele or PBP2 A501 amino acid substitutions, which decrease the susceptibility to ESCs,^1^^,^^32–35^ were found in 14.4% (*n *=* *33) and 65.1% (*n *=* *149) of all isolates, respectively. All isolates with a mosaic *penA* allele [*penA*-10.001 (*n *=* *20), *penA*-34.001 (*n *=* *11) and *penA*-92.001 (*n *=* *2)]^44^ were isolated in 2015–16. The majority (75.8%) of these isolates clustered in the phylogenomic tree and belonged to MLST ST7363 (*n *=* *16) and ST1901 (*n *=* *9). The amino acid substitution in PBP2 A501 (*n *=* *149) was A501T (*n *=* *73) or A501V (*n *=* *76). A501T was always in combination with PBP2 G542S (*n *=* *70) or PBP2 P551L (*n *=* *3), while isolates with A501V were less frequent in combination with G542S (*n *=* *2) or P551S (*n *=* *21). AMR mutations in *mtrR* (increasing antimicrobial efflux through the MtrCDE efflux pump), *penB* (decreasing antimicrobial intake through PorB1b) and *ponA* [encoding PBP1 (L421P), associated with penicillin resistance]^1^^,^^29^^,^^31–35^ were found in 79.0% (*n *=* *181; 87.0% in 2011 and 71.9% in 2015–16), 88.2% (*n *=* *202; 84.3% in 2011 and 91.7% in 2015–16) and 94.8% (*n *=* *217; 95.4% in 2011 and 94.2% in 2015–16), respectively, of all isolates. Furthermore, two isolates from 2011 had an identical mosaic *mtrD* sequence causing increased antimicrobial MtrCDE efflux and non-WT to azithromycin (MIC = 2 mg/L and MIC = 4 mg/L), and the MtrD sequence differed by only four amino acid alterations compared with some previously described mosaic MtrD sequences^45^^,^^46^ (Figure 1). No isolates contained any target determinants for azithromycin resistance in 23S rRNA or for spectinomycin resistance in 16S rRNA or the *rpsE* gene. Fluoroquinolone resistance mutations in *gyrA* (GyrA S91) were found in 99.1% (*n *=* *227; 98.1% in 2011 and 100.0% in 2015–16) and in *parC* in 90.8% (*n *=* *208; 89.8% in 2011 and 91.7% in 2015–16) of isolates. The *rpsJ* V57M mutation, involved in tetracycline resistance, was found in 98.7% (*n *=* *226) of isolates. Finally, *tetM*-carrying plasmids and *bla*_TEM_-carrying β-lactamase plasmids were found in 42.4% (*n *=* *97; 50.0% in 2011 and 35.5% in 2015–16) and 28.4% (*n *=* *65; 25.9% in 2011 and 30.6% in 2015–16) isolates, respectively.

**Figure 2.**
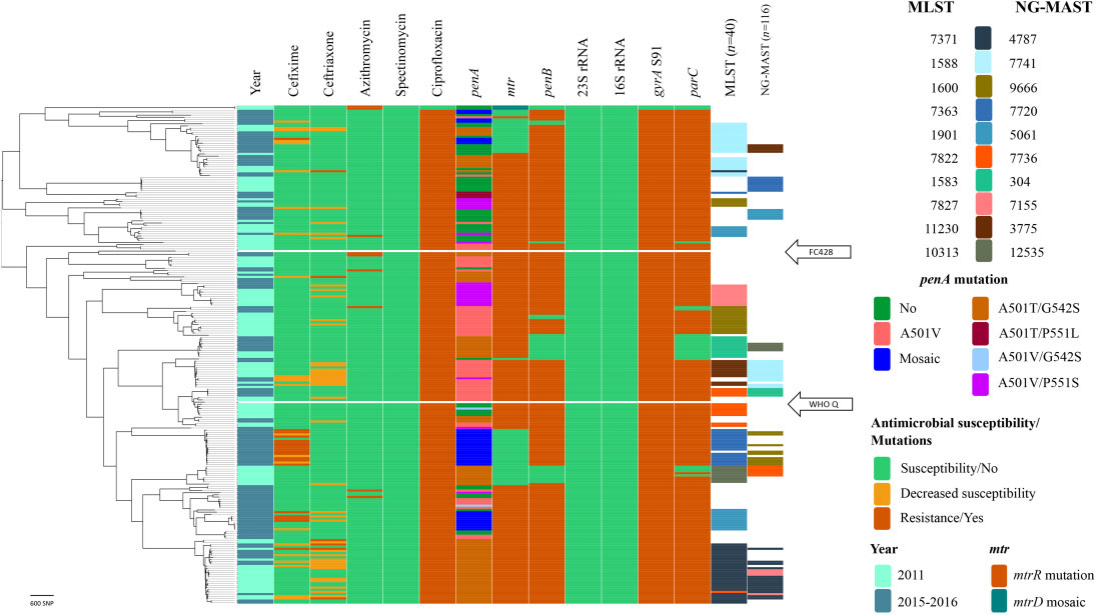
Phylogenomic tree with susceptibility to the most therapeutically relevant antimicrobials, AMR determinants, the most common MLST STs and *N. gonorrhoeae* multiantigen sequence typing (NG-MAST) STs for *N. gonorrhoeae* isolates from Vietnam in 2011 (*n *=* *108) and 2015–16 (*n *=* *121). The WHO Q^20–22^ reference strain and FC428^10^ are included for comparison and are marked with white bars. This figure appears in colour in the online version of *JAC* and in black and white in the print version of *JAC*.

**Figure 3.**
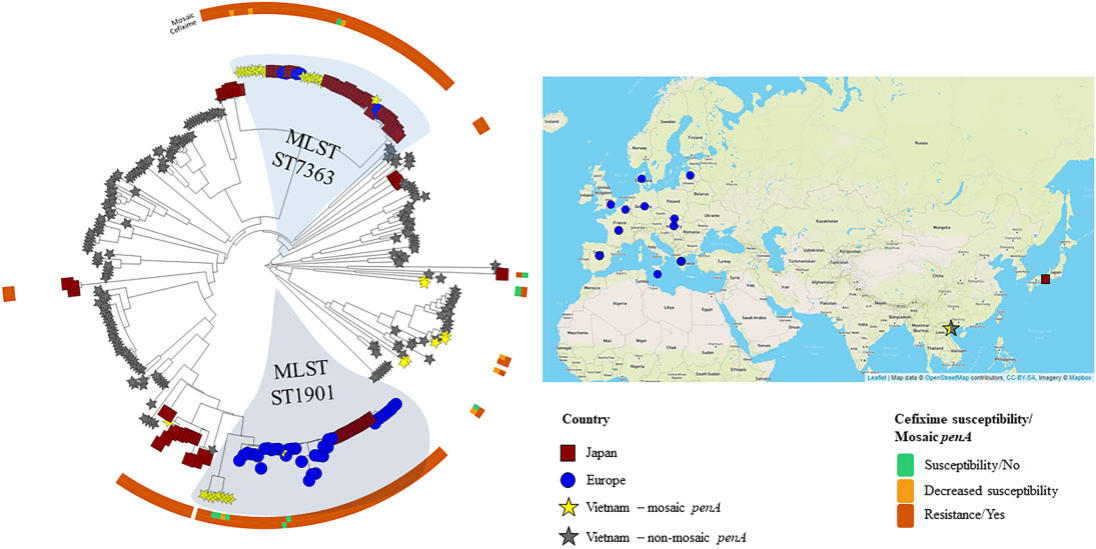
Circular phylogenomic tree visualized using Microreact^42^ of all isolates from Vietnam (*n *=* *229) compared with cefixime-resistant isolates (MIC >0.125 mg/L) from Japan (*n *=* *69)^37^ and 11 European countries (*n *=* *42).^32^ Coloured bars represent cefixime susceptibility and the presence of the mosaic *penA* allele. This figure appears in colour in the online version of *JAC* and in black and white in the print version of *JAC*.

In Figure 2, the phylogenomic structure and phenotypic AMR for all isolates (*n *=* *229; 108 in 2011 and 121 in 2015–16), including their AMR determinants, for the therapeutically most relevant antimicrobials are illustrated. Two new clades in 2015–16 [42 (18.3%) of the 229 isolates] included the majority (94.4%) of the cefixime-resistant isolates and most (78.8%) of the isolates with a mosaic *penA* allele. No isolate genomically similar to the FC428 strain^10^ was found. However, a minor clade of isolates (*n *=* *8) from 2011 had relatively close relationships to WHO Q (Figure 1),^20–22^ differing by 137–195 SNPs. The Vietnamese isolates carrying mosaic *penA*, mostly MLST ST7363 and ST1901, were related to cefixime-resistant isolates spreading in Japan in 2015 (mostly with mosaic *penA*-10)^37^ and in 11 European countries in 2013 (mostly NG-MAST genogroup 1407 with mosaic *penA*-34)^32^ (Figure 3).

### Concordance of resistance phenotype and genotype for the therapeutically most relevant antimicrobials

All ciprofloxacin-resistant isolates (*n *=* *227; 99.1%) had the main fluoroquinolone resistance determinant, i.e. the *gyrA* S91F mutation, and all ciprofloxacin-susceptible isolates (*n *=* *2; 0.9%) were lacking mutations in GyrA amino acid positions S91 and D95. Eighteen (94.7%) of the cefixime-resistant isolates (*n *=* *19; 8.3%) had a mosaic *penA* allele [*penA-*10.001 (*n *=* *14) and *penA-*34.001 (*n *=* *4)] and the remaining cefixime-resistant isolate had a non-mosaic *penA-*18.001 encoding a PBP2 with A501T plus G542S mutations. Nevertheless, eight (3.5%) additional isolates had a mosaic *penA* allele [*penA-*34.001 (*n *=* *5) and *penA-*10.001 (*n *=* *3)], but were susceptible to cefixime (MICs = 0.032–0.064 mg/L). All six ceftriaxone-resistant isolates contained *penA-*18.001, encoding PBP2 with A501T and G542S amino acid alterations, *mtrR* and *penB* AMR determinants, although belonging to different clades in the tree. However, 27 other isolates with PBP2 A501T plus G542S amino acid alterations, *mtrR* and *penB* AMR determinants remained ceftriaxone susceptible. Finally, none of the nine (3.9%) azithromycin-non-WT isolates had any macrolide resistance-associated mutation in the 23S rRNA gene (A2059G or C2611T). However, two closely related isolates from 2011 had identical mosaic *mtrD* genes (Figure 1) and the remaining seven azithromycin-non-WT isolates had the characteristic A deletion in the *mtrR* promoter sequence (Figure 2), both causing an overexpression of the MtrCDE efflux pump.

## Discussion

Our findings show that cefixime, azithromycin, benzylpenicillin, tetracycline and ciprofloxacin should not be used for empirical first-line monotherapy in Vietnam as in most other countries globally.^1–3^ Nevertheless, in Vietnam and many additional countries, particularly (but not exclusively) in South-East and East Asia, enhanced surveillance of gonococcal AMR, antimicrobial stewardship and molecular epidemiology are crucial.

The decrease in ceftriaxone-resistant gonococcal isolates from 2011 (5%) to 2015–16 (1%) in Vietnam is promising. However, the level of cefixime-resistant isolates increased from 1% to 15%, which was mainly due to the introduction and clonal expansion of two cefixime-resistant gonococcal mosaic *penA-*carrying clones that were related to cefixime-resistant strains spreading in Japan^37^ and Europe^32^ for many years. Cefixime has been frequently used for gonorrhoea treatment in Vietnam,^27^ which has probably facilitated the dissemination of these cefixime-resistant strains. Worryingly, these mosaic *penA* alleles only need one SNP to also develop high-level resistance to ceftriaxone. Of the 2015–16 isolates (*n *=* *121), 18 (14.9%) were resistant to cefixime and all contained a mosaic *penA* allele, which affects the MIC of cefixime more than the MIC of ceftriaxone. In contrast, all six ceftriaxone-resistant isolates (4.6% in 2011 and 0.8% in 2015–16) had a non-mosaic *penA* allele encoding a PBP2 with A501T and G542S amino acid alterations, which together with overexpression of the MtrCDE efflux pump and decreased ESC intake through PorB significantly increases the MIC of ceftriaxone in particular. Furthermore, despite low resistance (1%) to ceftriaxone in 2015–16, 12.4% (*n *=* *15) of the 2015–16 isolates had a decreased susceptibility to ceftriaxone and all these isolates contained PBP2 with an A501 (80.0%) alteration or a mosaic *penA* allele (20%). All isolates were susceptible to spectinomycin, and it would be valuable to have spectinomycin widely available internationally again.

WGS is very valuable for molecular epidemiology (emergence, transmission and evolution of strains and their AMR), nationally and internationally, and for detection of AMR determinants to predict AMR.^29–37^ For *N. gonorrhoeae*, prediction of ciprofloxacin resistance can be effectively conducted by detection of mutations in GyrA codon S91. Furthermore, it can be important to survey for specific *penA* alleles, ideally in combination with ceftriaxone and cefixime susceptibility data. However, despite the fact that most gonococcal isolates with cefixime resistance harbour a mosaic *penA* allele, many gonococcal isolates with mosaic *penA* alleles do not show *in vitro* and/or clinical resistance to cefixime.^1^^,^^32–35^ In the present study, no mutations in the azithromycin resistance determinant 23S rRNA were found, but 3% and 5% of the isolates from 2011 and 2015–16, respectively, were non-WT to azithromycin. This resistance might be due to overexpression of the MtrCDE efflux pump in particular, as shown, but also to additional unknown macrolide resistance mechanisms. Using a more comprehensive geographical, temporal and genomically diverse collection of gonococcal isolates could improve surveillance strategies and AMR prediction based on WGS. It was recently reported that real-time WGS using the small hand-held MinION can rapidly and relatively accurately sequence genomes and predict resistance/non-WT to ciprofloxacin and azithromycin, and decreased susceptibility to ESCs in gonococcal isolates.^47^ Accordingly, rapid and reliable prediction of gonococcal AMR using WGS might be available in routine diagnostic laboratories in the near future.

In Vietnam, the recommended treatment for uncomplicated gonorrhoea is 400 mg of cefixime, 250 mg of ceftriaxone, 2 g of spectinomycin or 1 g of cefotaxime plus, and all these are given together with 200 mg of doxycycline daily for 7 days.^28^ Nevertheless, the compliance with these treatment recommendations in the public and, in particular, the private sector is unknown and other antimicrobials and doses are also used for monotherapy of some patients. First-line empirical dual therapy with at a minimum 500 mg of ceftriaxone combined with azithromycin should be considered in Vietnam and many additional countries in South-East and East Asia.

The main limitations of this study included the relatively low number of gonococcal isolates which were only from the capital city Hanoi, the lack of epidemiological data concerning the patients (e.g. identification of risk groups such as MSM and place of infection) and the low number of isolates from females.

In conclusion, from 2011 to 2015–16 an increased resistance to all currently and previously used antimicrobials except ceftriaxone, spectinomycin and tetracycline was identified among the heterogeneous *N. gonorrhoeae* isolates obtained in Vietnam. After 2011, cefixime resistance has rapidly increased in Vietnam, which was shown to be due to the introduction and subsequent clonal expansion of two mosaic *penA*-containing strains, which were genomically similar to cefixime-resistant gonococcal strains spreading in Europe^32^ and Japan^37^ for many years. Furthermore, a minor clade (eight isolates) relatively similar to the XDR strain WHO Q was identified; however, these isolates were not resistant to ceftriaxone due to the lack of mosaic *penA*-60. Isolates similar to WHO Q have also been identified in, for example, China and Japan,^21^ and, for improved understanding of emergence and spread of ceftriaxone resistance, it is important to identify the origin of such XDR strains. Continued and significantly expanded surveillance of gonococcal AMR and molecular epidemiology, using WGS, and improved antimicrobial stewardship in Vietnam and other Asian countries are crucial.

## Supplementary Material

dkaa040_Supplementary_Data

## Supplementary data

Figure S1 is available as Supplementary data at *JAC* Online.
