# Supplementary figures and images for: Genomic analysis and antimicrobial resistance of Neisseria gonorrhoeae isolates from Vietnam in 2011 and 2015–16
Source: J Antimicrob Chemother. 2020 Feb 18;75(6):1432–8. doi: 10.1093/jac/dkaa040 (PMC7382555; doi:10.1093/jac/dkaa040)

**Supplementary data**

**Figure S1**


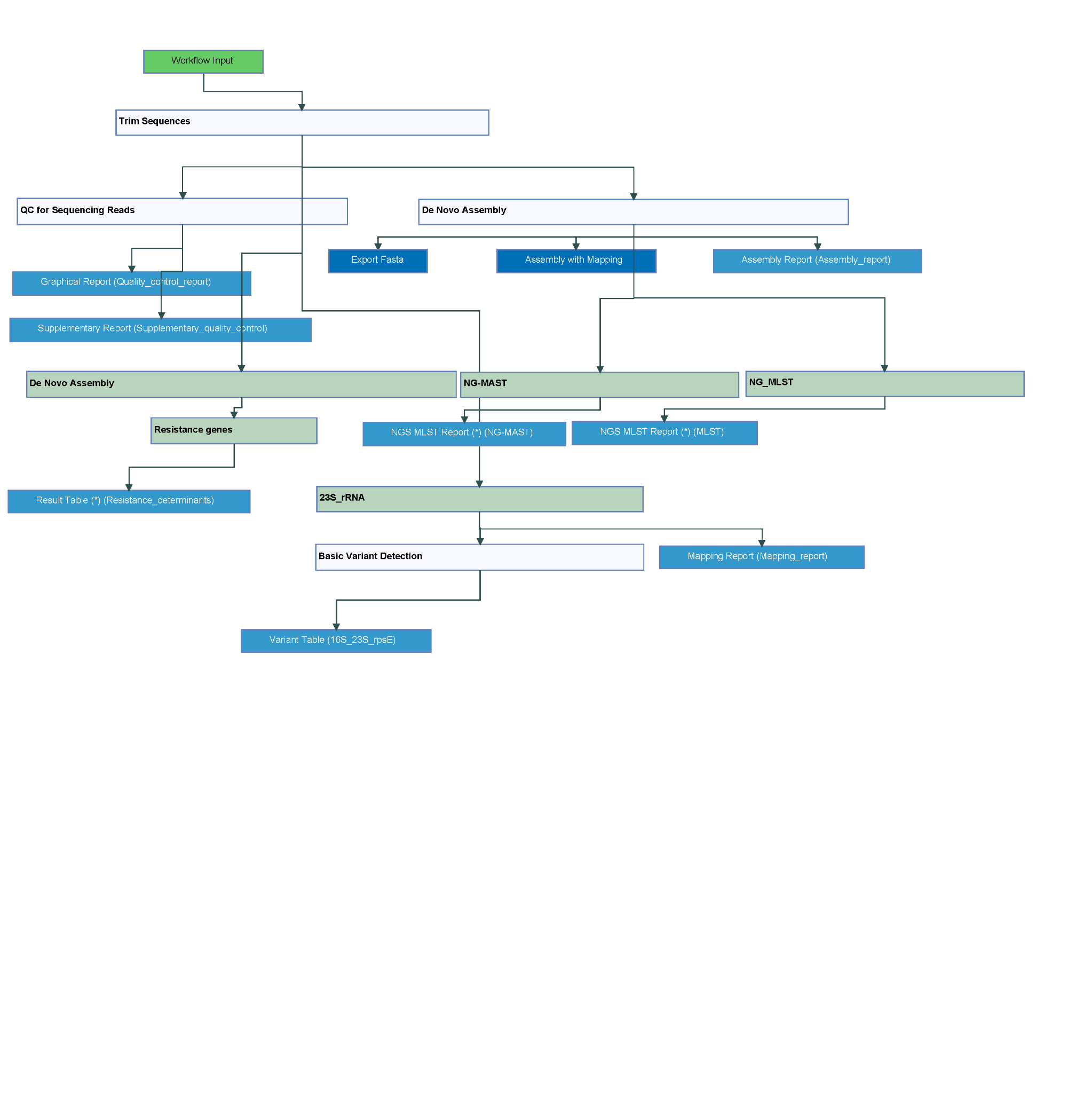

Supplement: dkaa040_Supplementary_Data [file dkaa040_supplementary_data.docx]
